# *IGF-I* and *GH* Genes polymorphism and their association with milk yields, composition and reproductive performance in Holstein–Friesian dairy cattle

**DOI:** 10.1186/s12917-024-04188-4

**Published:** 2024-08-02

**Authors:** Ahmed A. Saleh, Tarek G. M. Hassan, Dalia K. A. EL-Hedainy, Adel S. A. El-Barbary, Mahmoud A. Sharaby, Elsayed E. Hafez, Amr M. A. Rashad

**Affiliations:** 1 Animal and Fish Production Department, Faculty of Agriculture (Al-Shatby), Alexandria University, Aflaton St, Alexandria City, 11865 Egypt; 2 Arid Lands Cultivation Research Institute, City of Scientific Research and Technological Applications, Alexandria, New Borg El Arab, 21934 Egypt

**Keywords:** *IGF-I*, *GH*, Holstein–Friesian, RFLP, SNP, DNA sequencing

## Abstract

**Background:**

The insulin-like growth factor (*IGF-I*) and growth hormone (*GH*) genes have been identified as major regulators of milk yield and composition, and reproductive performance in cattle. Genetic variations/polymorphism in these genes have been found to influence milk production, yield and quality. This investigation aimed to explore the association between *IGF-I* and *GH* polymorphisms and milk yield and composition, and reproductive performance in a herd consisting of 1000 Holstein–Friesian (HF) dairy cattle from *El-Alamia* farm. The experimental animals were 76 ± 7.25 months in age, with an average live weight of 750 ± 50.49 kg, and raised under the same conditions of feeding and weather. The studied animals were divided into three categories; high producers (*n* = 280), medium producers (*n* = 318) and low producers (*n* = 402).

**Results:**

The digestion of 249 bp for *IGF-I-SnaBI* using the Restriction-fragment-length-polymorphism (RFLP) technique yielded two alleles; T (0.59) and C (0.41) and three genotypes; TT (0.52), TC (0.39) and CC (0.09) and this agrees with the results of DNA/gene sequencing technique. The sequencing analysis of the *IGF-I* gene revealed polymorphism in position 472 (C > T). Nucleotide sequencing of the amplified fragment of the *IGF-I* gene of different genotypes was done and submitted to the NCBI GenBank with Accession no. MH156812.1 and MH156811.1. While the digestion of 432 bp for *GH-AluI* using the RFLP technique yielded two alleles; A (0.81) and G (0.19) and two genotypes; AA (0.77) and AG (0.23) and this agrees with the results of DNA/gene sequencing technique. The sequencing analysis of the *GH* gene revealed polymorphism in the position 1758 C > G and in turn led to changes in amino acid sequence as Alanine for (A) compared to Glycine for (G). Nucleotide sequencing of the amplified fragment of the *GH* gene was done and submitted to the NCBI GenBank with Accession no. MH156810.1. The results of this study demonstrate the effects of variants of the *GH-IGF-I* somatotrophic axis on milk production and composition traits in commercial HF cattle. The greatest values of milk yield and reproductive performance were observed on *IGF-I-SnaBI-TC* and *GH-AluI-AG* genotypes. While the greatest % fat and % protein values were observed on *IGF-I-SnaBI-CC* and *GH-AluI-AA* genotyped individuals.

**Conclusion:**

The genetic variation of the studied genes can be utilized in selecting animals with superior milk yield, composition and reproductive performance in Holstein–Friesian Dairy Cattle under subtropical conditions.

**Supplementary Information:**

The online version contains supplementary material available at 10.1186/s12917-024-04188-4.

## Background

With the advancement of technologies and molecular genetics, livestock breeding has become more efficient and cost-effective [1, 2]. In the last few decades, exploring the role of major/ candidate genes which control livestock production has become a focus of research [3]. For instance, the genetic regulation of insulin-like growth factor-I (*IGF-I*) [4] and growth hormone (*GH*) [5] genes have been well-studied in many livestock species, including cattle [5], sheep [6], camels [7] and pigs [8]. The molecular genetic studies of *IGF-I* and *GH* genes mainly targets their association with growth and reproductive performances, carcass traits, and milk yield and composition [5, 9].

The *IGF* growth factor system consists of two ligands (*IGF-I* and *IGF-II*) and two cell-surface receptor types *I* and *II*. *IGF-I* and *II* are structurally related proteins, playing a key role in cell differentiation, growth, embryogenesis, regulation of metabolism and regulation of cell proliferation [10, 11]. *IGF-I* gene encodes a hormone similar in structure to insulin, which controls cell growth and differentiation. It, also called somatomedin C, is a protein that in humans is encoded by the *IGF-I* gene [12]. The *IGF-I* gene is located on chromosome 12 at position 12q23 in humans, on 5 in cattle, on 6 in pigs and 10 in mice [13]. *IGF-I* is a polypeptide of a molecular weight 7.5 kDa built of 70 amino acids. It is produced primarily by the liver as an endocrine hormone as well as in target tissues in a paracrine/autocrine fashion. In dairy cattle, *IGF-I* acts primarily on the mammary gland to stimulate milk synthesis [12–15]. This hormone also affects milk composition, including fat and protein content [16]. In the liver, *IGF-I* is dynamically regulated by lactation and energy balance [17]. Moreover, *IGF-I* plays an essential role in pre-and postnatal growth, muscle development, and bone formation [11]. The promoter region of the *IGF-I* gene contains several single nucleotide polymorphisms (SNPs), which regulate transcriptional activity and association with growth performance [7]. Studies have shown that several *IGF-I*-SNPs were associated with growth performance, carcass traits, and milk yield in dairy cattle [18–20]. Cattle with specific *IGF-I*-SNPs have superior growth performance and higher milk yield [9, 21].

On the other hand, the *GH* gene is located on chromosome 20 in cattle and on chromosome 6 in pigs. It is a single-chain polypeptide of approximately 22 kDa, composed of 190 or 191 amino acids. The *GH* gene encodes the *GH* pituitary-derived hormone [22–24] that plays a vital role in animal physiology. It stimulates growth, milk production, and animal reproduction in livestock [25, 26]. Studies have shown that certain *GH* gene polymorphisms were related to milk production traits [27, 28]. For instance, a variant of the *GH* gene called the A allele is associated with higher milk production in Holstein cows [28]. Several investigations reported that specific *GH*-SNPs have increased feed efficiency and growth rates [20, 29]. In cattle, a SNP in the *GH* gene was associated with a carcass weight increase of 4 kg and a 2.3% increase in feed efficiency. Similarly, pigs with the GG genotype of *GH* had a significant increase in feed efficiency and reduced backfat thickness. [20, 29].

*IGF-I* is a primary mediator of the effects of *GH*. The action of *GH* is mediated by the transmembrane *GH-R* [14]. The binding of *GH* to G*H-R* activates an intracellular signalling pathway that induces the transcription of many genes including the *IGF-I* gene. *GH* is made in the anterior pituitary gland, released into the bloodstream, and then stimulates the liver to produce *IGF-I* which in turn stimulates systemic body growth through inducing growth-promoting effects on almost every cell in the body, especially skeletal muscle, cartilage, bone, liver, kidney, nerves, skin, hematopoietic cells and lungs [18, 30]. In addition to the insulin-like effects, *IGF-I* can also regulate cell growth and development, especially in nerve cells, as well as cellular DNA synthesis. The *GH-IGF-I* system controls processes, such as; fertility, lactation milk and nutrient partitioning necessary for lactogenesis [30].

Both *GH* and *IGF-I* play a crucial role in animal growth and development [19]. There is an intricate relationship between *GH* and *IGF-I* as the former stimulates hepatic *IGF-I* synthesis [19, 31]. *IGF-I*, in turn, positively feedbacks on *GH* secretion and modulates its receptor expression. Moreover, *IGF-I* affects animal reproduction, lactation, and feed intake by interacting with the somatotropic axis [32–34]. Both genes have been shown to play critical roles in milk production and composition in dairy cattle, with variations in these genes affecting milk output and quality [23, 25, 26]. By understanding how these genes function and interact, researchers can work to optimize milk production and improve the quality of their dairy products [18, 24]. Therefore, understanding the genetic regulation and interaction between the *GH* and *IGF-I* genes is essential in animal breeding programs.

In this aspect, according to the studies which aimed at enhancing mastitis resistance through selection, improving mastitis-related genetic traits in cattle is imperative. Somatic cell count (SCC) is an essential trait in such selection studies and is known to have positive genetic correlations with mastitis ranging from 0.36 to 0.67 [35]. Reduction of milk SCC through proposed selection studies not only aids in improving mastitis resistance but also helps reduce mastitis incidence [36]. Conversely, mastitis significantly alters the ion composition of milk, leading to increased electrical conductivity (EC), sodium, and chloride contents, as well as elevated milk pH resulting from the mixing of blood and extracellular fluid components in inflamed quarters with secreted milk [37]. In identifying and managing these traits, milk SCC, EC, and pH-related genes or polymorphisms need to be identified and improved accordingly [38]. In dairy cows, SCC exhibited positive correlation with clinical mastitis and with body weight [39]. As such, *IGF-I* and *GH* polymorphisms may also be explored for their strength of correlations with SCC, EC or pH [40, 41].

Although *IGF-I* genotypes have been extensively studied, little is known about their impact on blood composition in dairy cows during the periparturient period, which spans before and after calving [29]. However, *IGF-I* concentrations are altered during the postpartum period exhibiting linkage to various reproductive aspects [24, 42]. To address this knowledge gap, a part of the current study was conducted to investigate the relationship between *IGF-I* gene polymorphisms and its concentration in the periparturient period of Holstein–Friesian (HF) dairy cows.

Few investigations are available on *GH-IGF-I* polymorphisms and their association with milk yield and composition, and reproductive performance in HF cattle under subtropical conditions. To the best of our knowledge, no comprehensive investigations have examined differences in both *GH* and *IGF-I* genes in association with milk traits. Thus, the present study aimed to; 1) investigate the association between the polymorphism in *IGF-I* gene and milk yield and composition, and reproductive performance in HF cattle. 2) Explore the relationship between different *GH* genotypes and milk yield and composition, and reproductive performance in HF cattle. 3) examine the association between *IGF-I* gene polymorphisms and its concentration in the periparturient period of HF dairy cows.

## Results

**Fig. 1:**
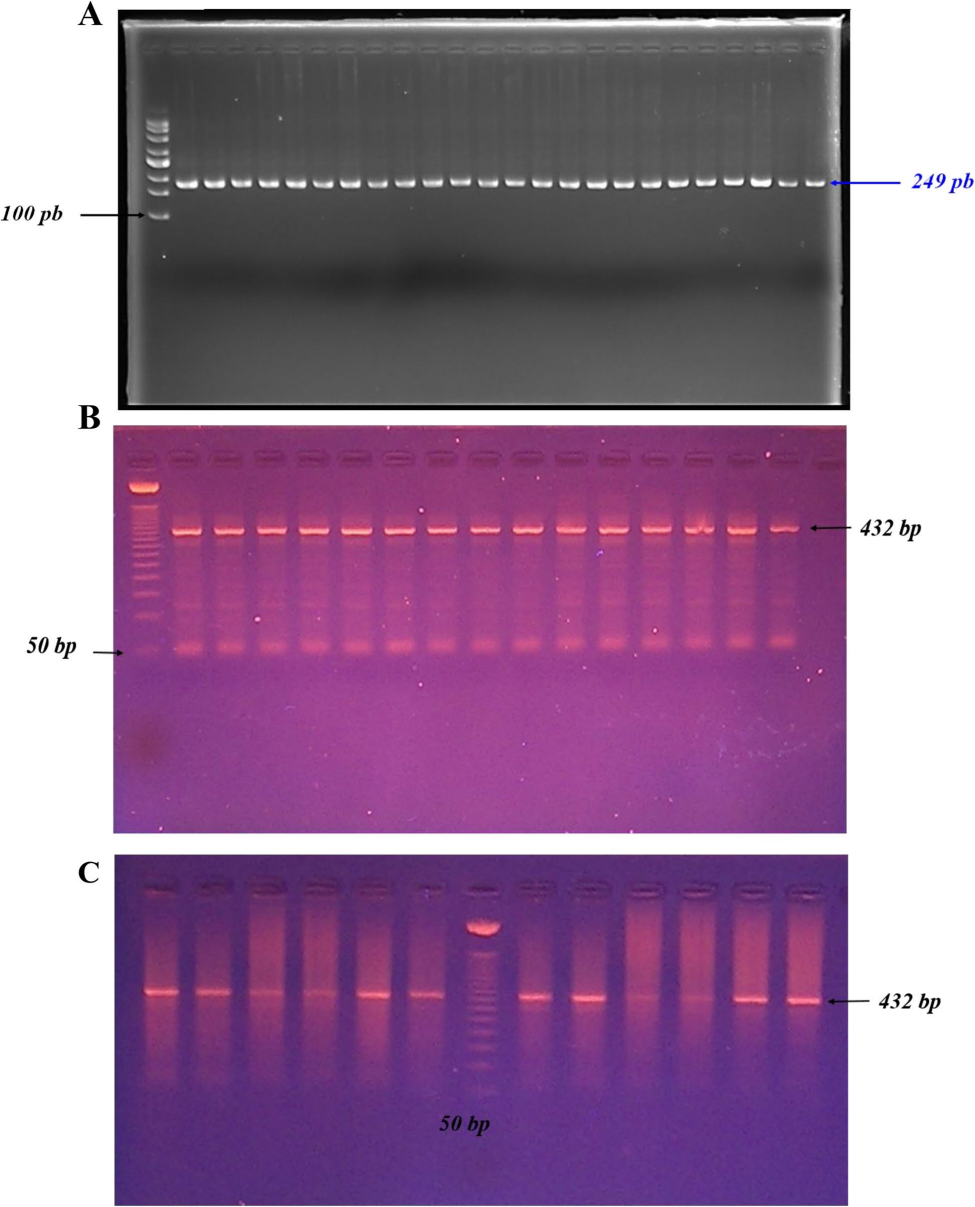
**A** PCR amplification of the 5’-noncoding region of the bovine *IGF-I* gene from Holstein Frisian cattle. M, 50 bp DNA ladder. **B** PCR amplification of *GH* gene fragment from Holstein Frisian cattle. M, 100 bp DNA ladder. **C** Purified PCR product of *GH* gene (432 bp) from Holstein Frisian cattle. M, 50 bp DNA ladder

This study concerns mainly the polymorphism among tested cows for *IGF-I* and *GH* genes. It also spotlights the relationship between the differentiation of *IGF-I* and *GH* genes and milk yield and composition, and reproductive performance. PCR amplification for the tested animals produced an amplified 249 bp fragment for the *IGF-I* gene (Fig. 1A and Fig. S1A), and 432 bp for the *GH* gene (Fig. 1B, C and Fig. S1B).

### Insulin-like Growth Factor -1 (*IGF-I*)

#### PCR amplification and genotyping of IGF-I gene by RFLP

**Fig. 2:**
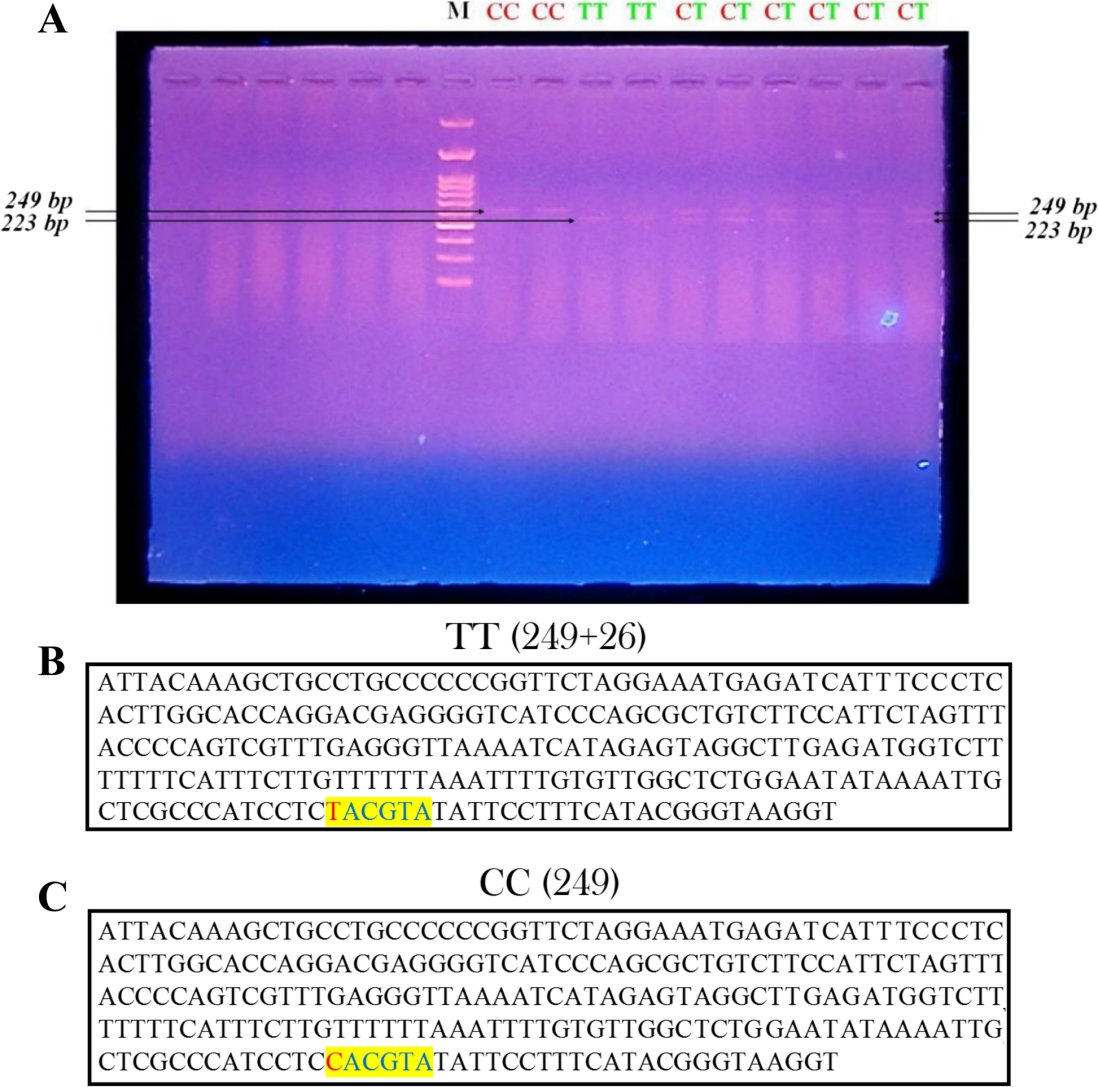
**A** Agarose gel electrophoresis showing RFLP-*SnaBI* restriction pattern of Holstein Frisian cattle in 5’-noncoding region of the bovine *IGF-I* gene Lane M: 50-bp ladder marker. Lanes 1 and 2: Homozygous CC; genotype non-digested PCR product (249 bp). Lanes 3 and 4: Homozygous TT genotype with 2 restricted fragments at 223 and 26 bp. Lanes 5 and 6: heterozygote CT genotype with 3 restricted fragments at 249, 223 and 26 bp. The restriction fragment with size 26 bp has not been seen on the gel. **B** Result of endonuclease restriction with *SnaBI* using *FastPCR* C/T: Single nucleotide substitution, genotype TT, and **C** genotype CC

The amplified 249 bp fragment of HF cows of the *IGF-I* gene contains the 5’-noncoding region of the bovine *IGF-I* gene (Fig. 1A). A **C** → **T** substitution in the gene creates a new *SnaBI* restriction site, allowing for analysis using RFLP techniques. The PCR products obtained from HF cows were digested with *SnaBI*, resulting in three patterns: **1)** Homozygous (CC) genotype with a non-digested PCR product (249 bp).** 2)** Homozygous (TT) genotype with 2 restricted fragments at 223 and 26 bp. **3)** Heterozygote (CT) genotype with 3 restricted fragments at 249, 223, and 26 bp. The 26 bp restriction fragment was not observed on the gel (Fig. 2A).

#### Sequence analysis and frequencies

The three genotypes in tested animals due to a restriction site at position 472 (TAC^GTA). Worth mentioning that this particular position (P^472^) has been previously identified in various cattle breeds [43, 44]. In the present study, the frequencies of the *IGF-I*-*SnaBI* alleles T and C were 0.59 and 0.41, respectively (Table 1).

**Table 1:** Genotypic and allelic frequencies of *IGF-I*-*SnaBI* and *GH-AluI* genes, and diversity parameters for the 472 C > T of *IGF-I* gene and the 1758 C > G (Leucine /Valine) substitution of *GH* gene

The genotype of *IGF-I*-*SnaBI* gene									
**Holstein Friesian Groups**	N^*^	**Allele**		**Genotype**			***H***_***o***_	***H***_***E***_	***PIC***
		**T**^**1**^	**C**^**2**^	**TT**	**TC**	**CC**			
**High producer**	280	0.64	0.36	0.30 (85)	0.69 (193)	0.01 (2)	0.145	0.141	0.212
**Medium producer**	318	0.69	0.31	0.35 (110)	0.59 (188)	0.06 (20)	0.119	0.106	0.112
**Low producer**	402	0.82	0.18	0.80 (322)	0.03 (11)	0.17 (69)	0.093	0.089	0.107
**Total**	1000	0.59	0.41	0.52 (517)	0.39 (392)	0.09 (91)	–-	–-	–-
**Genotype of *GH*-*AluI* gene**									
**Holstein Friesian Groups**	N^*^	**Allele**		**Genotype**			***H***_***o***_	***H***_***E***_	***PIC***
		**A**^**3**^	**G**^**4**^	**AA**	**AG**	**GG**			
**High producer**	280	0.74	0.26	0.52 (145)	0.48 (135)	0.00	0.163	0.144	0.233
**Medium producer**	318	0.89	0.11	0.77 (245)	0.23 (73)	0.00	0.125	0.117	0.125
**Low producer**	402	0.98	0.02	0.96 (386)	0.04 (16)	0.00	0.083	0.081	0.134
**Total**	1000	0.81	0.19	0.77 (776)	0.23 (224)	0.00	–-	–-	–-

^*^N Sum of sample (head), *T*^*1*^ Thiamine base, *C*^*2*^ Cytosine base, ^*3*^*A* Alanine, ^4^*G* Glycine, *H*_*o*_ Heterozygosity, *H*_*E*_ Heterozygosity expected. *PIC* polymorphism information content

**Fig. 3:**
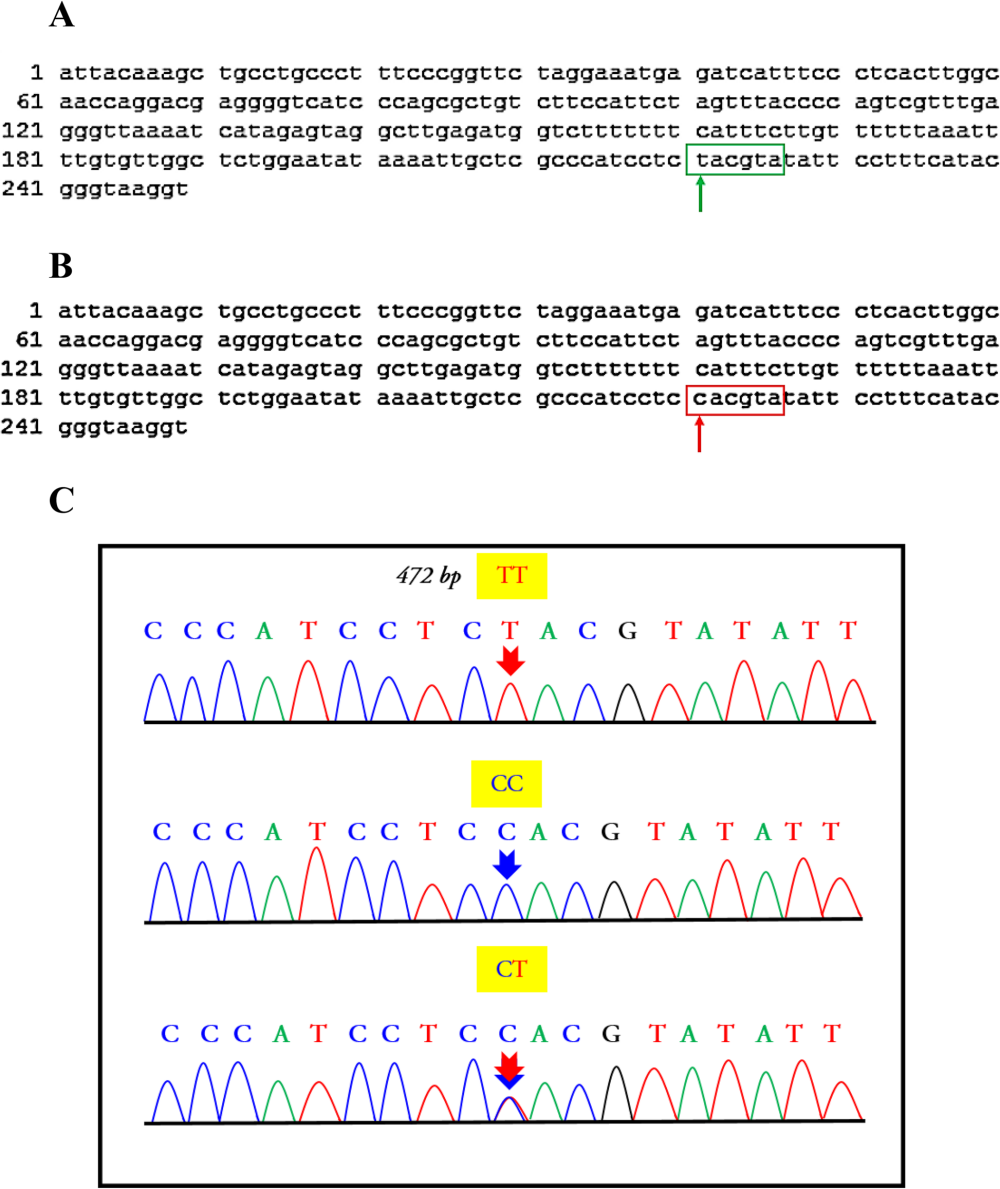
**A** A 249 bp sequence of *IGF-I* gene for Holstein Frisian genotype (AA = TT) (*NCBI accession no MH156812*). **B** a 249 bp sequence of *IGF-I* gene for Holstein Frisian genotype (CC) (*NCBI accession no. MH156811*). **C** The chromatogram of the sequenced 5’-noncoding region *IGF-I* gene showing homozygote (CC and TT) and heterozygote (CT) genotypes

Nucleotide sequencing of the amplified IGF-I gene fragment of HF cows was submitted to the NCBI GenBank (Accession no. MH156812.1 and MH156811.1) (Fig. 3A, B). The transition mutation (C > T) in the *IGF-I* gene was found with the detected three genotypes (Fig. 3C).

#### Serum periparturient concentration and their association with IGF-I genotypes

An evidence for a significant (*p* > 0.01) association between C and T mutations in position 472 of the *IGF-I* gene and its serum concentration in HF cows in Egypt was found. The highest serum concentration of *IGF-I* was found in CC genotype cows in which CC genotype had significantly higher (*p* > 0.01) concentration of *IGF-I* at 20 d prepartum (92.44 µg/L) compared to those with TT genotype (74.58 µg/L). Also, the values of *IGF-I* concentration for 20 d before calving and 50 d postpartum were higher (*p* > 0.01) in cows with CC genotype compared to those with TT genotype. The C/T transition for the trend of *IGF-I* concentration between the CC and TT genotype was significantly different (*p* > 0.01) during the 20 d prepartum and 50 d postpartum (Table 2).

**Table 2:** Comparison between the serum concentration (Mean ± SE)of different *IGF-I* (µg/L) genotypes in HF dairy cows

Traits	IGF-I-SnaBI polymorphism genotypes			*p*-value
	**TT (n = 100)**	TC (*n* = 100)	CC (*n* = 80)	
**Periparturient periods:**				
**20 days prepartum**	74.58 ± 6.17^c^	86.32 ± 2.01^cb^	92.44 ± 1.03^a^	*p* ≤ 0.01
**25 days postpartum**	16.63 ± 1.07^a^	16.99 ± 0.90^a^	16.04 ± 1.00^a^	*p* ≤ 0.01
**50 days postpartum**	24.45 ± 2.80^b^	28.23 ± 1.99^a^	29.33 ± 1.76^a^	*p* ≤ 0.01
**Overall mean**	38.56 ± 3.75^c^	43.47 ± 1.03^ab^	46.43 ± 2.09^a^	*p* ≤ 0.01

*SE* Standard Error

^a^^−^^c^ means that different superscript letters in the same row are different

### Growth hormone (*GH*) gene

#### PCR amplification of bovine GH gene

The fragment of 432 bp of the *GH* gene was successfully amplified from the genomic DNA of the tested cows (Fig. 1B &C). In the present study, the amplification of the *GH* gene produced a specific band of 432 bp and a nonspecific fragment of 120 bp (Fig. 1B). So, PCR purification from the gel was performed to isolate the specific fragment (Fig. 1C).

#### Genotyping of GH gene by RFLP and nucleotide and Protein sequence

**Fig. 4:**
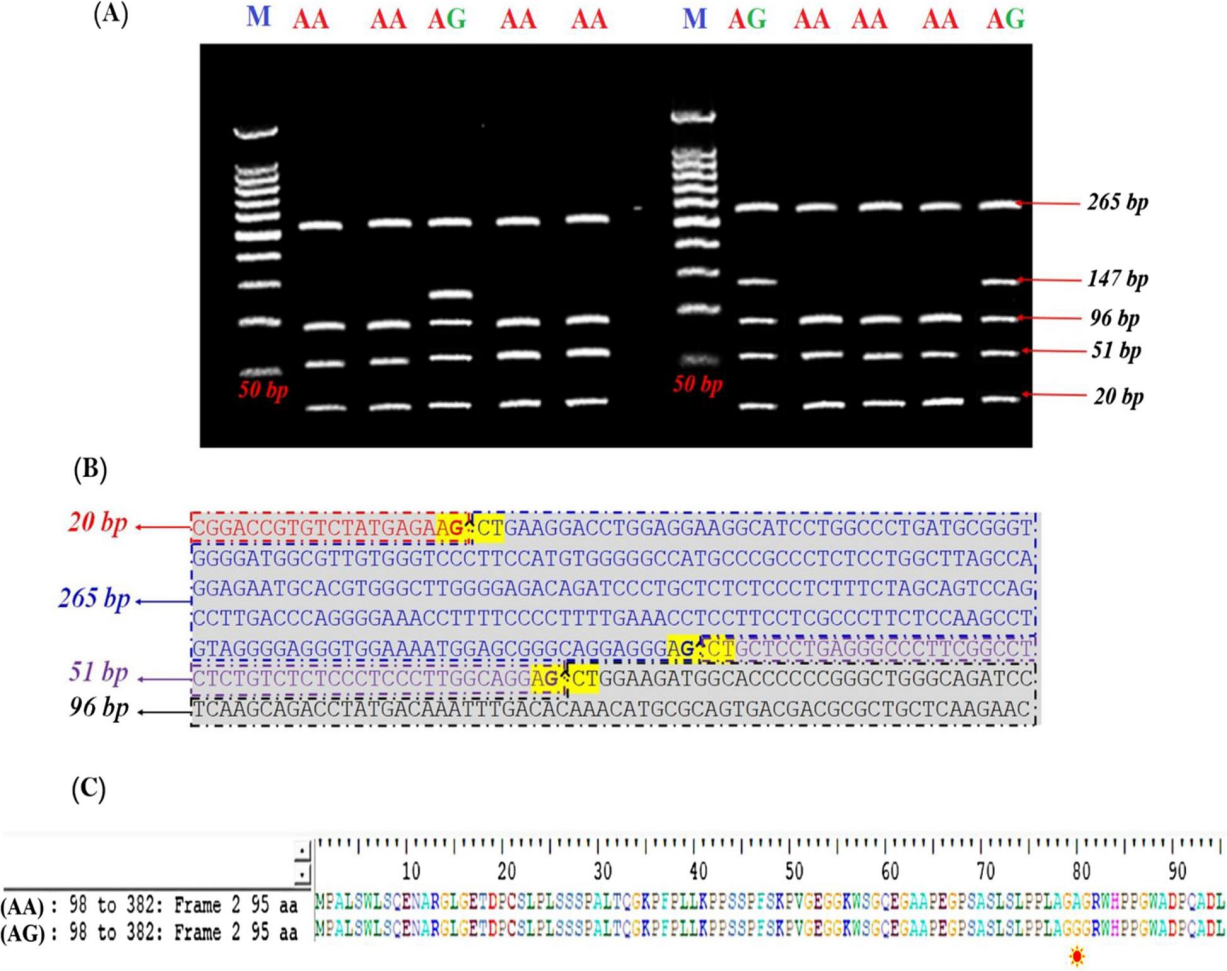
**A** Agarose gel electrophoresis showing RFLP-*AluI* restriction pattern of Holstein Frisian cattle in *GH* gene Lane M: 50-bp ladder marker. Lanes AA: Homozygous (AA) genotype with 4 restricted fragments at 265, 96, 51 and 20 bp. Lanes AG: heterozygote (AG) genotype with 5 restricted fragments at 265, 147,96, 51 and 20 bp. **B** A 432 bp sequence of *GH* gene for Holstein Frisian genotype (AA) (*NCBI accession no. MH156810.1*) with RFLP-*AluI* restriction pattern (4 restricted fragments at 265, 96, 51 and 20 bp) and restriction sites (AG^CT). **C** Amino acids comparison of amplified *GH* gene of tested Holstein Friesian using (*MEGA-11*) Molecular Evolutionary Genetics Analysis, and its site https://www.megasoftware.net

The site recognized by the *AluI* enzyme restriction was A**G**^**C**T bases. Three *AluI* restriction sites produced fragment lengths of 20, 147 and 265 bp, known as [G] allele (Glycine) and three *AluI* restriction sites that produced fragment lengths of 20, 51, 96 and 265 bp, known as [A] allele (Alanine). A heterozygous AG genotype was then identified by fragment lengths of 20, 51, 96, 147, and 265 bp. GG genotype was not present for all samples (Fig. 4 A and B). Genotyping results produced only two genotypes AA and AG, but not GG. The genotypes frequencies were 77 and 23% for AA and AG, respectively. The frequency of A allele was very high (81%) compared to that of G allele (19%) (Table 1).

**Fig. 5:**
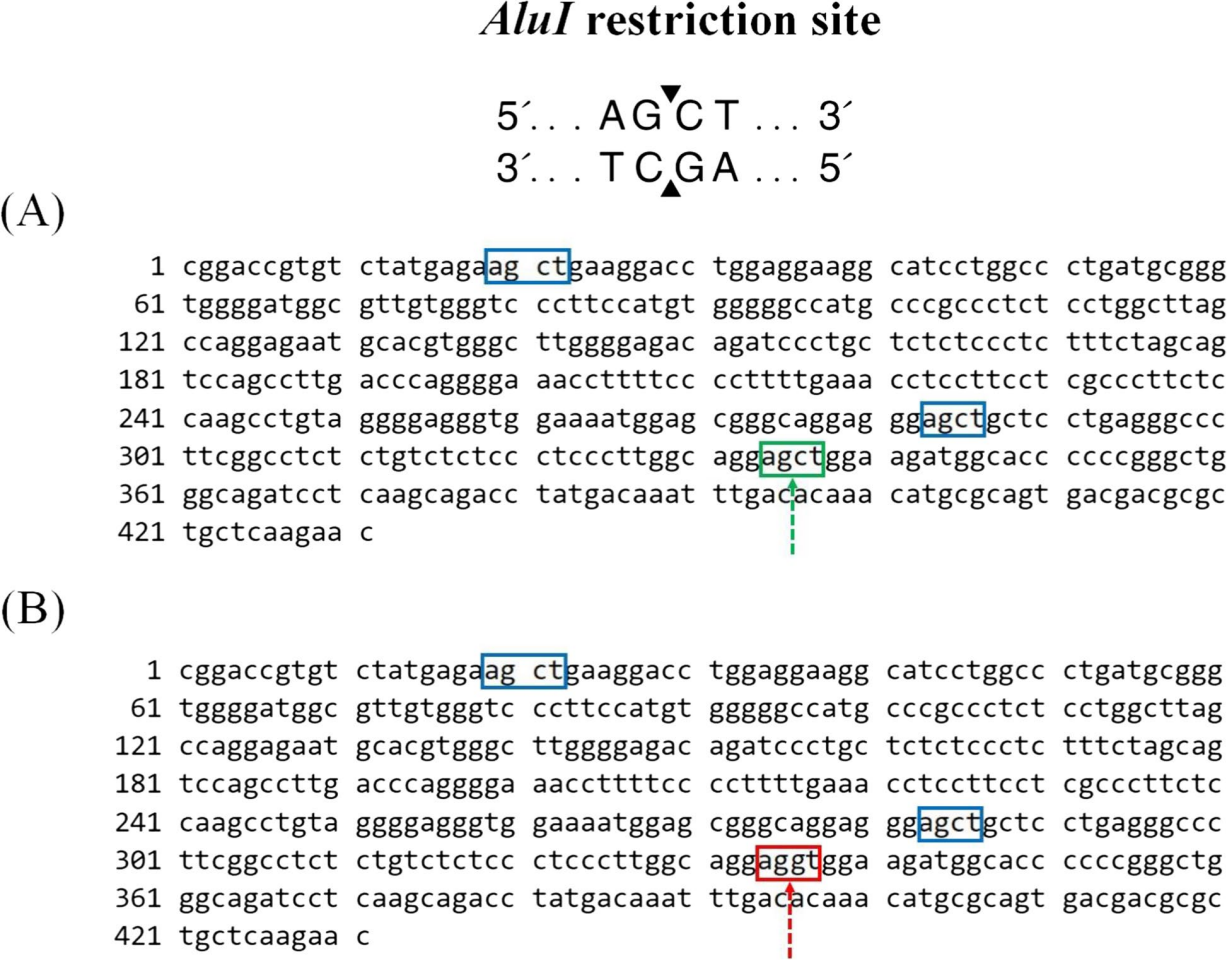
**A** A 432 bp sequence of *GH* gene for Holstein Frisian genotype (AA) (*NCBI accession no. MH156810.1*), with 4 restricted fragments at 265, 96, 51 and 20 bp. **B** A 432 bp sequence of *GH* gene for Holstein Frisian genotype (GG) with 3 restricted fragments at 20, 265 and 147

A 432 fragment from Intron 4, part of exon 4 and part of exon 5 was sequenced. Data was generated and manged by BioEdit V.7.7. (https://bioedit.software.informer.com/7.2) and GeneScan (http://hollywood.mit.edu/cgi-bin/genscanw_py.cgi) with a minimum ORF size of 20 and the start codon AGT, and the comparison of amino acids was done by using MEGA 11 (https://www.megasoftware.net). The amino acids sequence of genotype (AA) was different from that of genotype (AG) in one amino acid. The changed amino acid was Alanine for (AA) compared to Glycine for (AG) (Fig. 4C). Nucleotide sequencing of the amplified fragment of the bovine *GH* gene for (AA) genotype was done and submitted to the NCBI GenBank (Accession no. MH156810.1), (Fig. 5).

### The relationship between productive and reproductive performance and the prevalence of *IGF-I *and *GH* genotypes

#### Insulin-like Growth Factor -1 (IGF-I)

Table 3 shows the relationship between the *IGF-I*-*SnaBI* polymorphism genotypes and productive and reproduction traits. Concerning the *IGF-I* gene, there were three genotypes; TT (n = 517), TC (n = 392) and CC (n = 91). The TC genotype achieved the shortest (*p* > 0.01) LP and DPRY followed by CC and then TT genotypes, respectively. In this aspect, the TC genotype recorded the highest TMY and 305d-MY (*p* > 0.01) followed by TT and then CC. Regarding the percentage of fat and protein, CC genotype was the highest (*p* > 0.05) followed by TT and then TC. Also, CC genotype recorded the lowest value for EC followed by CT and then TT genotypes. There were no significant differences in SCC and pH among different genotypes of *IGF-I* gene (Table 3).

**Table 3:** Relationship of *IGF-I*-*SnaBI* genotypes polymorphism with productive and reproduction traits (Mean ± SE)

Traits	IGF-I-SnaBI-genotypes polymorphism			*p*-value
	TT (*n* = 517)	TC (*n* = 392)	CC (*n* = 91)	
**Productive performance:**				
**LP** (d)	404.47 ± 4.03^a^	321.79 ± 2.95^c^	343.53 ± 3.84^b^	*p* ≤ 0.01
**DPRY** (d)	68.31 ± 1.22^b^	61.50 ± 1.00^c^	73.32 ± 1.02^a^	*p* ≤ 0.01
**TMY** (d)	8027.46 ± 67.41^b^	9200.80 ± 44.17^a^	4329.01 ± 80.34^c^	*p* ≤ 0.01
**305d-MY** (kg)	5865.04 ± 33.80^b^	7974.54 ± 50.45^a^	3030.30 ± 34.12^c^	*p* ≤ 0.01
**Fat** (%)	2.40 ± 0.09^b^	1.34 ± 0.05^c^	3.82 ± 0.13^a^	*p* ≤ 0.01
**Protein** (%)	1.94 ± 0.04^b^	1.15 ± 0.08^c^	2.92 ± 0.09^a^	*p* ≤ 0.01
**SCC** (Log_10_SCC)	5.19 ± 0.011	5.11 ± 0.008	5.04 ± 0.009	*p* = 0.461
**pH**	6.87 ± 0.001	6.87 ± 0.001	6.88 ± 0.002	*p* = 0.801
**EC** (mS/cm)	5.15 ± 0.005	5.08 ± 0.009	5.00 ± 0.001	*p* = 0.541
**Reproductive performance:**				
**AFC** (mo)	28.87 ± 0.10	28.59 ± 0.11	28.56 ± 0.12	*p* = 0.741
**FPE** (d)	90.85 ± 0.91^b^	95.28 ± 1.32^a^	91.23 ± 1.07^b^	*p* ≤ 0.01
**NI**	2.82 ± 0.02	2.71 ± 0.05	2.70 ± 0.07	*p* = 0.751
**DOPN** (d)	226.27 ± 5.50^a^	139.38 ± 6.40^c^	211.95 ± 2.74^b^	*p* ≤ 0.01
**CI** (d)	481.09 ± 4.10^b^	397.28 ± 2.85c	503.51 ± 2.00^a^	*p* ≤ 0.01
**GL** (d)	276.79 ± 0.21	277.52 ± 0.11	277.35 ± 0.13	*p* = 0.661

*LP* Lactation Length, *DPRY* Dry period Length, *TMY* Total Milk Yield, *305d-MY* Adjusted Milk Yield, *% Fat* Fat Percentage, *% Protein* Protein Percentage, *SCC* Somatic cell count, *pH* Acidity *EC* Electrical Conductivity, *FPE* The First Postpartum Estrus, *NI* The Number of Inseminations, AFC Age at First Calving, *DOPN* Days Open, *CI* Calving Interval, *GL* Gestation length, *SE* Standard Error, *mo* Month, *d* Day

^a^^−^^c^means that different superscript letters in the same row are different

As for reproductive performance, the TT genotype recorded the shortest (*p* > 0.05) FPE compared to TC and CC which had non-significantly different FPE. On the other side, the shortest DOPN and CI were observed for TC genotype (*p* > 0.01) compared to TT and CC genotypes. However, no significant differences in AFC, NI and GL were observed among different genotypes of *IGF-I* gene.

In the current study, the novel detected SNPs and their amino acids sequence for *IGF-I* gene could be considered as candidate SNPs for milk yield and composition, and for reproductive performance in HF cows under the subtropical conditions of Egypt (Table 1). For instance, one SNP was detected for 472 C > T of *IGF-I* gene in the high-producing cows (with TC genotype), this sequence resulted in a significant differentiation in LP, DPRY, TMY and 305d-MY (*p* > 0.01) comparing to other sequences (Table 3).

#### Growth hormone (GH) gene

Table 4 shows the relationship between *GH*/*AluI* polymorphism genotypes and productive and reproduction traits. There were two genotypes; AA (*n* = 776) and AG (*n* = 224) for the *GH* gene of the studied cows. The cows with AG genotype recorded shorter (*p* > 0.05) LP and DPRY compared to AA genotype. In this aspect, AG genotype achieved the highest (*p* > 0.01) TMY and 305d-MY compared to AA. On the other side, AA recorded the highest (*p* > 0.01) percentage of fat and protein. Also, AA recorded the lowest value for EC compared to AG genotype. There were no significant differences in SCC and pH among different genotypes in the *GH* gene.

**Table 4:** Relationship between the *GH*/*AluI* genotypes polymorphism with productive and reproduction traits

Traits	*GH*/*AluI-*genotypes polymorphism		*p*-value
	**AA **(***n* = 776)**	**AG (*n* = 224)**	
**Productive performance:**			
** LP** (d)	342.63 ± 1.89^a^	322.12 ± 3.50^b^	*p* ≤ 0.01
** DPRY** (d)	74.14 ± 1.33^a^	62.20 ± 0.98^b^	*p* ≤ 0.01
** TMY** (kg)	4299.31 ± 77.15^b^	9210.60 ± 58.20^a^	*p* ≤ 0.01
** 305d-MY** (kg)	3028.15 ± 59.80^b^	7895.61 ± 39.18^a^	*p* ≤ 0.01
** Fat** (%)	3.91 ± 0.10^a^	1.41 ± 0.08^b^	*p* ≤ 0.01
** Protein** (%)	2.87 ± 0.09^a^	1.20 ± 0.05^b^	*p* ≤ 0.01
** SCC** (Log_10_SCC)	5.08 ± 0.02	4.95 ± 0.01	*p* = 0.431
** pH**	6.89 ± 0.002	6.87 ± 0.001	*p* = 0.811
** EC (**mS/cm)	4.99 ± 0.008	5.12 ± 0.006	*p* = 0.201
**Reproductive performance:**			
** FPE** (d)	91.53 ± 1.33^b^	94.74 ± 1.07^a^	*p* ≤ 0.01
** NI**	3.29 ± 0.12	3.23 ± 0.10	*p* = 0.431
** AFC** (mo)	29.36 ± 0.11	28.79 ± 0.09	*p* = 0.312
** DOPN** (d)	212.78 ± 4.50^a^	138.46 ± 3.76^b^	*p* ≤ 0.01
** CI** (d)	504.33 ± 4.75^a^	389.39 ± 2.99^b^	*p* ≤ 0.01
** GL** (d)	277.22 ± 0.21	277.61 ± 0.24	*p* = 0.892

*LP* lactation length, *DPRY* Dry period Length, *TMY* Total Milk Yield, *305d-MY* Adjusted Milk Yield, *% Fat* Fat Percentage, *% Protein* Protein Percentage, *SCC* Somatic cell count, *pH* Acidity *EC* Electrical Conductivity,* FPE* The First Postpartum Estrus, *NI* The Number of Inseminations, AFC Age at First Calving, *DOPN* Days Open, *CI* Calving Interval, *GL* Gestation length, *SE* Standard Error, *mo* Month, *d* Day

^a^^−^^b^ means that different superscript letters in the same row are different

Regarding reproductive performance, the AA recorded the shortest (*p* > 0.05) FPE compared to AG. On the other hand, the shortest DOPN and CI were observed on AG genotype (*p* > 0.01) compared to AA. There were no significant differences in AFC, NI and GL among different genotypes of *GH* gene.

Another novel detected SNP for 1758 C > G (Alanine / Glycine) substitution (Fig. 5) in *GH* gene of high-producing cows (with AG genotype) was a mutation which led to changes in the amino acid sequence; Glycine for (G) > Alanine for (A) (Fig. 4C). This sequence resulted in a significant differentiation in LP, DPRY, TMY and 305d-MY (*p* > 0.01) comparing to another different sequence (Table 4).

Table 5 presents the *P*-values for various factors influencing productive and reproductive traits in HF cows. *P*-values measure the probability of obtaining the observed data, or more extreme results if the null hypothesis (no association between the factor and the trait) is true. A *p*-value less than the predetermined significance level (0.05 or 0.01) suggests statistical significance, indicating a strong association between the respective factor and the traits under consideration. The *p*-values in Table 5 help assess the strength of the evidence supporting the relationship between each factor and the productive and reproductive traits of HF cows. Factors with low *p*-values indicate a higher degree of significance and emphasize their importance in influencing the cow's performance. On the other hand, factors with higher *p*-values suggest a weaker association or lack of statistically significant impact on the traits. Interpreting the *p*-values within the context of the specific factors examined is crucial to drawing meaningful conclusions. For example, if a particular factor related to *IGF-I* and *GH* genotypes exhibits a low *p*-value, it implies that it significantly affects both productive (e.g., milk production or composition) and reproductive (e.g., calving interval) traits. This finding could have significant implications for dairy farmers, as it highlights the importance of considering this factor in their breeding or management strategies. Conversely, factors with high *P*-values may not have a substantial impact on productive and reproductive traits.

**Table 5:** The Significance levels of the factors affecting productive and reproductive traits (*P*-Values) of Holstein Friesian cows

Traits	DMP	Parity	Calving Year	Calving Season	Sire
**Productive performance:**					
** LP (**d**)**	< 0.001	< 0.001	< 0.001	< 0.001	< 0.001
** DPRY (**d**)**	< 0.001	0.019	< 0.001	0.052	< 0.001
** TMY (**kg**)**	< 0.001	0.035	< 0.001	0.004	< 0.001
** 305d-MY (**kg**)**	< 0.001	< 0.001	< 0.001	0.278	< 0.001
** Fat (**%**)**	< 0.001	0.159	< 0.001	< 0.001	0.014
** Protein (**%**)**	< 0.001	0.191	< 0.001	< 0.001	0.066
** SCC (**Log_10_SCC**)**	< 0.001	0.221	< 0.001	< 0.001	0.049
** pH**	< 0.001	0.181	< 0.001	< 0.001	0.037
** EC** (mS/cm**)**	< 0.001	0.122	< 0.001	< 0.001	0.024
**Reproductive performance:**					
** FPE** (d)	< 0.001	< 0.001	< 0.001	< 0.001	< 0.001
** NI**	< 0.001	< 0.001	< 0.001	< 0.001	< 0.001
** DOPN** (d)	< 0.001	< 0.001	< 0.001	< 0.001	< 0.001
** CI** (d)	< 0.001	< 0.001	< 0.001	< 0.001	< 0.001
** GL** (d)	0.196	0.344	0.001	0.172	0.001

*DMP* Daily milk production,* LP* lactation length, *DPRY* Dry period Length, *TMY* Total Milk Yield, *305d-MY* Adjusted Milk Yield, *% Fat* Fat Percentage, *% Protein* Protein Percentage, *SCC* Somatic cell count,* pH* Acidity, *EC* electrical conductivity, *FPE* the first postpartum estrus, *NI* The Number of Inseminations, AFC Age at First Calving, *DOPN* Days Open, *CI* Calving Interval, *GL* Gestation length, *mo* Month, *d* Day

## Discussion

The implementation of genomics offers a great opportunity for dairy cattle production improvement through increased genetic progress and inclusion of new traits of economic importance in the selection programs. The identification of potential SNPs in selected groups will make the exploitation of novel genomic selection methodology in farm animals possible. Better genetic characterization of breeds under different conditions/environments can help increase selection intensity and decrease generation interval [22, 24].

Studying the polymorphism in *IGF-I* and *GH* genes are important for the dairy cattle industry as they are involved in productive and productive traits [24]. There is evidence suggesting that specific variants in these two genes may possess either positive or negative associations with milk production, reproductive performance, and growth rate in dairy cattle in addition to some other species [22, 24]. Identifying polymorphisms should aid farmers and animal breeders in selecting stocks with advantageous gene variants for rapid improvement in productivity and, hence, profitability [15, 45, 46]. Additionally, verification of the genetic basis underlying the inheritance of these traits should help in developing targeted management strategies that maximize the dairy herd performance [20]. Therefore, exploring the polymorphism in *IGF-I* and *GH* genes is crucial for the development of the modern dairy industry.

*IGF-I* gene encodes a hormone similar in structure to insulin (Fig. S3), while, *GH* gene encodes *GH* hormone (Fig. S4). GH hormone, produced in the anterior pituitary gland, stimulates the release of *IGF-I* hormone from the liver and is of critical importance in the control of partitioning and nutrient utilization for growth, fertility, lactogenesis, and some necessary processes like development in mammalian [14, 33] (Fig. S5).

### Insulin-like Growth Factor -1 (*IGF-I*)

In the present study, polymorphisms among the studied HF cows for *IGF-I and GH* genes were detected. Also, an association of SNPs in the 5’-noncoding region of the *IGF-I* gene and the intron 4, part of exon 4 and part of exon 5 of the *GH* gene with the selected traits of milk production and composition, and reproductive performance in HF cows under subtropical conditions of Egypt were revealed.

Moreover, three genotypes for *IGF-I* gene (TT, TC and CC) were uncovered in the tested HF cows. Cows with the TC genotype were more productive for most of the milk yield traits, unlike the milk composition traits in which the CC genotype recorded the highest percentages for fat and protein in the milk (Table 3). This agrees with the results of Siadkowska et al. [47] obtained on 662 Polish-HF cows when examining the association between *IGF-I* gene polymorphism and the traits of feed intake, meat production, growth rate, and milk production. The heterozygous TC genotype yielded more fat-corrected milk, value-corrected milk, milk fat, and milk protein. Also, Silveira et al. [18] working on HF cows, Czerniawska-Piątkowska et al. [48] working on Holstein and Jersey cows [20] working on Madura cattle and Hartanto et al. [29] working on Jawa-Brebes cows, and obtained differentiated milk yield and composition and reproductive performance in response to different *IGF-I* and *GH* genotypes.

The effects of *IGF-I* gene polymorphism on cattle production, growth performance and developmental processes including metabolism and nutrient partitioning have been well-established and documented [9, 49]. The polymorphisms of *IGF-I* gene were first reported in Angus cattle in 1997 using the SSCP technique, and then identified as a C/T transition at position (P^472^) relative to the start of the transcription site at position 512 bp upstream from the ATG codon; according to the GenBank sequence (AF210383) [43].

The SNP of *IGF-I* gene described in the current study was similar to that found in the subtropical two strains of Nyalawi and Metairie cows [44], also, was in agreement with that advocated in *Bos taurus,* according to the *GenBank* (AF404761 and KF202095). In the same aspect, Mullen et al. [14] reported that nine SNPs were identified across a panel of twenty- two dairy and beef cattle by sequence analysis of the 5′ promoter, 3′ regulatory regions, intronic and encompassing 5 kb of *IGF-I* gene. Also, several SNPs were identified in the 3′ region of *IGF-I* and were associated (*p* < 0.05) with chest width and functional survival. On the other side, four out of nine SNPs were identified for their association with protein and fat yield, milk fat concentration, SCC, carcass conformation, and carcass fat (*p* < 0.05). These findings side by side with the results of the present investigation show strong effects of *IGF-I* polymorphism on milk production, fat yield and functional survival in cattle.

In the current study, the observed frequencies of different genotypes of *IGF-I* gene in HF cattle were (TT = 0.52), (TC = 0.39) and (CC = 0.09). This was in agreement with the results of Yazdanpanah et al. [50] who reported three frequencies of genotypes; (TT = 0.83), (TC = 0.14) and (CC = 0.03) on Najdi cattle (n = 84). Also, Nicolini et al. [31] confirmed that the frequencies of *IGF-I* genotypes for HF cows (*n* = 70) were (TT = 0.31), (TC = 0.54) and (CC = 0.14).

On the contrary, Nicolini et al. [31] reported that there was no effect of the different *IGF-I* genotypes on body condition change. In this regard, Omer et al. [44] reported no differences between two strains of Baggara zebu cattle at position 472 C > T of the *IGF-I* gene promoter. The mutant homozygote (TT) was detected in the Mesairi cattle only with a frequency of 0.016. While the heterozygote (CT) genotype existed with low allele frequencies (0.079 and 0.068) in Mesairi and Nyalawi breeds respectively. Also, Szewczuk et al. [51] reported that there was no association between *IGF-I*/*SnaBI* and dairy production traits in Polish Holstein cattle.

Concerning reproductive traits, this study revealed that *IGF-I*/*SnaBI* of TT genotype differed significantly (*p* < 0.05) from that of TC for FPE, whereas *IGF-I*/*SnaBI* of TC genotype had significantly shorter DOPN and CI (*p* < 0.01) compared to the other genotypes. Additionally, there were no significant differences observed among different genotypes of *IGF-I* gene for AFC, NI, and GL.

Concerning *IGF-I* concentration in the blood, an association was found between different *IGF-I* genotypes and the blood *IGF-I* concentration in HF cows. The findings of this study provide compelling evidence for a significant association between mutations in position 472 of the *IGF-I* gene and its serum concentration in HF cows in Egypt. Specifically, the CC genotype was found to have the highest serum concentration of *IGF-I*, with significantly higher levels compared to the TT genotype cows at 20 d prepartum. The data also showed that the CC genotype cows had higher *IGF-I* concentrations both 20 d before calving and 50 d postpartum compared to the TT genotype cows. Furthermore, the C/T transition for the trend of *IGF-I* concentration between the CC and TT genotypes was significantly different during the 20 d prepartum and 50 d postpartum. These results are summarized in Table 2, which illustrates the comparison between the serum concentrations of different *IGF-I* genotypes in HF dairy cows during periparturient periods. Overall, these findings indicate a strong link between the *IGF-I* gene mutations and serum concentration, highlighting the potential role of genetic factors in regulating *IGF-I* levels in HF cows.

Briefly, the highest serum concentration of *IGF-I* was found in CC followed by CT as compared to TT genotypes (Table 2). This is in agreement with the results of Mirzaei et al. [52] who confirmed that the highest serum concentration of *IGF-I* in Polish HF cows was found in CC followed by CT and then TT genotypes, which also, was in agreement with the reports of Mehmannavaz et al. [34], Bonakdar et al. [53] and Mirzaei et al. [52] on the Iranian Holstein cattle. In this regard, Gobikrushanth et al. [54] conducted a study to investigate the factors associated with the serum concentration of *IGF-I* in dairy cows and its relationship with reproductive outcomes. The study involved 647 lactating Holstein cows and identified various factors, such as herd, age, parity, pre-calving body condition score, and season of blood sampling, that influenced serum *IGF-I* concentrations. The researchers found that serum *IGF-I* concentration during the first week postpartum was higher in cyclic multiparous cows compared to acyclic ones, but did not show a significant association with ovarian cyclicity status in primiparous cows. The study also established optimal serum *IGF-I* thresholds predictive of pregnancy to first artificial insemination (P/AI) for primiparous and multiparous cows. Primiparous cows with high *IGF-I* had greater odds of P/AI and a tendency for higher pregnancy risk up to 150 d postpartum compared to those with low *IGF-I*. Similarly, multiparous cows with high *IGF-I* had increased odds of P/AI. Additionally, the researchers identified multiple SNPs associated with variation in serum *IGF-I* concentration, some of which were in linkage disequilibrium with candidate genes related to fertility.

On the other side, Wathes et al. [55] investigated the connection between negative energy balance (EB) and immune defense in peripartum dairy cows. The study found that cows with lower *IGF-I* levels, indicating poor EB, experienced more health problems, altered leukocyte functionality, and reduced milk production. In contrast, cows with higher *IGF-I* levels exhibited better immune function and milk production.

### Growth hormone (*GH*) gene

Internationally, three different genotypes for *GH*-*AluI* were detected in Iranian-Holstein cattle [27, 56]. In the present study, only two genotypes for the *GH* gene (AA and AG) were discovered in the studied cows. This is in agreement with the results of Kiyici et al. [27] on Holstein dairy cattle and with Pereira et al. [26], Curi et al. [57] and Misrianti et al. [28] who obtained only two genotypes in Brazilian Canchim, Brazilian Zebu and HF dairy cattle, respectively.

In the present investigation, the frequencies of genotypes were determined to be 77% and 23% for AA and AG, respectively. Notably, the A allele displayed a pronounced prevalence of 81% in contrast to the G allele, which exhibited a relatively modest frequency of 19% (Table 1). In this aspect, several studies have investigated the frequencies of genotypes and alleles of* GH* gene in different populations Lucy et al. [58] found variable frequencies of the two alleles across breeds. For HF cows, the frequencies were 0.93 and 0.07, for Brown Swiss were 1 and 0, for Jersey were 0.56 and 0.44, for Guernsey were 0.92 and 0.08, and for Ayrshire they were 0.79 and 0.21, respectively. In Holstein sires used for Artificial Insemination (AI), the frequencies of A and G alleles were 0.96 and 0.04. In another study, Kovacs et al. [59] observed genotypic frequencies of 87.05%, 12.40%, and 0.55% for AA, AG and GG genotypes in Hungarian-Holstein–Friesian (HHF). Dario et al. [60] reported 61%, 22%, and 17% frequencies for AG, AA and GG genotypes, respectively. Balogh et al. [61] found that the frequencies of AA, AG and GG genotypes were 83%, 17% and 0% in a specific population of cows. Hadi et al. [62] reported that the frequencies of AG, AA and GG genotypes were 61%, 39%, and 0%, respectively. They also observed that the A allele had a higher frequency (69%) compared to the G allele (31%).

In the current study, intron 4, part of exon 4 and part of exon 5 have been investigated for the *GH* gene. Previous researchers have identified polymorphisms in the 3rd and 4th introns, and 5th exon and the promoter of the *GH* gene in cattle. Also, recent reports show a significant relationship between polymorphisms in the *GH* gene and lactation performance in cattle [14, 24, 27].

This investigation revealed a genetic variation found between A and G alleles, due to mutation at the 1758 base resulting in the changing base from C to G (Fig. 5). Where the homozygous (AA) genotype was with 4 restricted fragments at 265, 96, 51 and 20 bp. While the heterozygote (AG) genotype was with 5 restricted fragments at 265, 147, 96, 51 and 20 bp (Fig. 4A). These findings are similar to the results of Misrianti et al. [28].

In the present investigation, cows with the *GH*-*AluI*-AG genotype were more productive for most of the milk yield traits unlike milk composition traits, where the *GH*-*AluI*-AA genotype recorded the highest percentages for fat and protein in milk (Table 4). This agrees with the results of Kovacs et al. [59] who confirmed that AG genotype showed to be advantageous for 305d-MY, while AA genotype recorded the highest percentages for fat and protein. Also, the current findings are in agreement with the results of Nugroho et al. [20] on Madura cattle who reported that the *GH*-*AluI*-AA genotype was discovered to have higher performance in milk fat and protein content, body and carcass weights compared the *GH*-*AluI*-AG genotype, while the *GH*-*AluI*-AG genotype was found to have higher performance in milk yield compared to *GH*-*AluI*-AA genotype. Moreover, Yardibi et al. [63] reported that the variant genotypes; AA, AG and GG of the *GH*-*AluI* gene had a positive correlation with percentages of fat and protein contents of milk. On the contrary, Shaidullin [64] confirmed that the highest level of milk productivity was found in animals with *GH*-*AluI* of AA genotype with a significant advantage over full-aged cows, *GH*-*AluI* of AG and *GH*-*AluI* of GG in terms of milk yield of 280 kg (*p* < 0.001) and 509 kg (*p* < 0.001), by the amount of milk fat of 9.1 kg (*p* < 0.001) and 18.5 kg (*p* < 0.01), by the amount of milk protein of 7.9 kg (*p* < 0.01) and 14.8 kg (*P* < 0.01).

In the present study, the heterozygous AG genotype exhibited changes in the amino acid sequence in position (P^114^) as Glycine replaced Alanine in AA genotype. In this regard, Lucy et al. [58] and Lucy et al. [30] reported that cytosine (C) substituted guanine (G) at position 2141 causing an amino acid change from Alanine to Glycine at residue 127 of the *GH* polypeptide. Moreover, the associations between milk production traits and Glycine (G) allele have been confirmed [27, 49, 59]. On the other hand, a substitution favouring Alanine (A) allele was achieved [64] in several cattle breeds.

Concerning reproductive performance, the *GH*-*AluI*-AA genotype showed differences in FPE (*p* < 0.05) compared to the *GH*-*AluI*-AG genotype. However, the latter had significantly shorter DOPN and CI (*p* < 0.01) compared to the *GH*-*AluI*-AA genotype. Additionally, no significant differences were observed among different genotypes of the *GH* gene for AFC, NI and GL. This agrees with the results of Amiri et al. [65] who reported that the individuals with the *GH*-*AluI*-AG genotype had significantly shorter DOPN and CI (*p* < 0.01) compared to the *GH*-*AluI*-AA genotype.

However, the results are contradictory with the studies by Lechniak et al. [66] and Lechniak et al. [25] who did not detect any significant relationship between *GH*-*AluI* gene polymorphism and reproductive performance especially for bulls’ sperm characteristics or parameters of *in-vitro* fertilization and embryo development.

Briefly, the greatest TMY and 305d-MY values and the best reproductive performance were observed on *IGF-I*-*SnaBI*-TC and *GH*-*AluI*-AG genotyped cows. While the greatest % fat and % protein values were observed on *IGF-I*-*SnaBI*-CC and *GH*-*AluI*-AA genotyped cows (Tables; 3 and 4).

On the other side, Lucy et al. [58] revealed that dairy cows having small mature size like Jersey breed had high frequency of *GH*-G allele, and those having large mature size like Holstein breed had high frequency of* GH*-A allele. Otherwise, Balogh et al. [61] reported that animals carrying genotype AG were prone to higher basal insulin levels (*p* = 0.064), a longer time to reach half of the maximal and basal insulin concentrations (*p* = 0.035 and *p* = 0.054, respectively) and larger insulin area under the curve (*p* = 0.032). Expanding on this relationship, Mullen et al. [67] confirmed that there was an association between *GH* genotypes and carcass traits as well as SCC and body condition score (BCS). Also, Mullen et al. [14] found an association between genetic variation in *GH* gene and fertility, pregnancy rate and overall pregnancy rate. Moreover, Hadi et al. [62] reported that *GH*-*AluI*-AA genotype reduced dystocia, compared to *GH*-*AluI*-AG. Overall, these studies shed light on the intricate associations between *GH* genotypes and various phenotypic traits, including size, insulin levels, carcass traits, fertility, and dystocia.

### *GH-IGF-I* system controls

*IGF-I* and *GH* genotypes are of significant importance when it comes to milk production and quality as well as fertility performance in HF dairy cattle. The somatotrophic axis, which includes *GH* and *IGF-I*, plays a crucial role in regulating growth and development in cattle, affecting traits such as milk yield, growth rate, body composition, and fertility (Fig. S5).

In this regard, reliable studies confirmed that the somatotrophic axis, which essentially consists of growth hormone-releasing hormone (*GHRH*), *GH*, IGF-*I* and *II* and their associated binding proteins (*GHBP*, *IGFBP1-6*) and receptors (*GHRHR*, *GHR*, *IGF-IR* and *IGF-IIR*), plays a key role in the metabolism and physiology of mammalian growth [21, 68]. The somatotrophic axis (*GH-IGF*) is a key regulator of animal growth and development and affects performance traits that include milk yield, growth rate, body composition, and fertility [14, 19, 23], (Fig. S6). *GH* and *IGF-I* are major regulators of postnatal metabolism, growth and consequently play critical roles in the control of mammary gland development, lactation, growth processes, and fertility in cattle [30, 69]. The actions of *GH* vary significantly in several physiological states [70], but the net effect of this hormone throughout early lactation supports a helpful role for the indirect actions of *GH* on lipolysis and gluconeogenesis [11] and attenuated growth-promoting actions and support by *IGF-I* in peripheral tissues [71]. Within the dairy cow, the per-parturient reduction in *IGF-I* synthesis is related to a concomitant reduction in the liver-specific *GH* receptor type 1A (*GHR1A*) [72].

In light of the above facts and findings in the present investigations, understanding the impact of *IGF-I* and *GH* genotypes on milk production, quality, and fertility in HF dairy cattle, especially under subtropical conditions in Egypt is essential.

## Conclusions

In conclusion, studying *IGF-I* and *GH* genes has shed light on their roles in livestock growth and development. The genetic regulation of these genes has increased the efficiency of selecting superior-value animals in meat, production, and reproduction programs. The molecular genetic studies of *IGF-I* and *GH* genes have demonstrated their potential for livestock improvement. Greatest milk yield and composition values, and reproductive performance were observed on *IGF-I*-*SnaBI*-TC and *GH*-*AluI*-AG genotyped individuals. While the greatest % fat and % protein values were observed on *IGF-I*-*SnaBI*-CC and *GH*-*AluI*-AA genotyped individuals. The genetic variation of these genes can be utilized in selecting animals with superior milk yield, growth performance, feed efficiency and meat quality. Continued research in the area of genetic regulation of these genes is necessary to further explore their roles in livestock breeding and production.

## Methods

### Animals and Sampling

The present investigation is part of a project aiming to assess HF cattle (n = 1000) under subtropical conditions (Egypt) in order to aid the characterization of cattle genetic resources and genome analysis in this area for milk yield and composition, and reproductive performance.

A total of 1000 HF dairy cattle from *El-Alamia* commercial dairy farm (*belonging to Universal Company for Agricultural Development and Soil Reclamation, herd located at Nubaria region in the K 90 Alex-Cairo desert road, Egypt*) (Fig. S7) were investigated for milk production and composition, and reproductive performance.

### Management of animals

The calving period for the tested animals was constrained between January 2016 and January 2018. The experimental cows were 76 ± 7.25 months in age, with an average live weight of 750 ± 50.49 kg, randomly selected from the respective groups of contemporaries born within 7–11 months. Cows were housed free in open semi-shaded yards, nourished under the prevailing feeding conditions. The cows were fed according to the INRA feeding system for ruminants on a complete Total Mixed Ration (TMR) diet consisting mainly of wilted grass silage, corn silage, beet pulp, cotton seed, soybean, barley, and concentrate mix and vitamin mixture, supplemented with minerals. Water was available excessively. The cows were raised under consistent conditions of nutrition and weather, and milked twice daily at 07:00 and 16:00 h and were classified according to milk yield, specifically the daily milk yield (DMY) into; high producer cows (n = 280) with a DMY above 35 kg, medium producer cows (n = 318) with a DMY between 25 ~ 35 kg, and low producer cows (n = 402) with a DMY below 25 kg.

### Traits of concern

The traits of concern were; 1) Lactation characteristics: lactation length (LP), dry period length (DPRY), total milk yield (TMY) and adjusted milk yield (305d-MY). 2) Milk composition: fat percentage (% fat) and protein percentage (% protein). 3) Milk Quality; somatic cell count (SCC), electrical conductivity (EC) and acidity (pH). 4) Reproductive performance: days open (DOPN), calving interval (CI), gestation length (GL), the first postpartum estrus (FPE) and the number of inseminations (NI). These traits were recorded professionally for the studied cows for several lactations from the 1st to 4th. 5) Serum concentration of ***IGF-I*** (µg/L) for different milk yield/groups of HF dairy cows.

### Genes of concern

In the current study, the polymorphism in *IGF-I* and *GH* genes of HF cows were investigated for their association with milk yield and composition, and reproductive performance of cattle.

### Milk samples

Two 50 ml milk samples were taken monthly on a specific test day from the morning (07:00 am) and evening milkings (4:00 pm) of each cow and were kept at 4 °C until used to determine milk composition until the fourth month of lactation. A total of 2770 milk samples were obtained and utilized during the 4th lactation of cows.

Milk SCC measurements were taken with the help of The NucleoCounter®-SCC-100™ equipment (Chemometec, Bohemia, New York, USA), while, EC and pH measurements were taken with the help of Milkana Multi-Test milk analyser (http://mtm-solutions.com/en/products/detail/Milkana-MULTI---TEST).

### Blood samples

#### Blood samples for genetic analysis

A blood sample of 5 ml from venous blood was collected separately from the jugular vein of each tested cow using venojects. Blood samples were treated with 0.5 ml of 2.7% EDTA (Pspark, U.K), as an anticoagulant, kept in an icebox and transferred immediately to the lab and stored at -80 °C up to the genetic analysis. All procedures carried out with the use of animals were approved by the Ethics Commission, permission No. AU082211211117), Faculty of Agriculture (*Al-Shatby*), Alexandria University, Egypt.

#### Blood samples for serum IGF-I concentration

Also, a total of 280 healthy cows from the three different *IGF-I* genotypes TT (n = 100), TC (n = 100) and CC (n = 80) were chosen randomly to measure periparturient serum *IGF-I* concentration. The blood samples were collected from the jugular vein of each cow into a separate tube (Guangzhou Improve Medical Tech. Co. Ltd., China) without anticoagulants for biochemical indices calculation. The blood samples have been taken 20 days before the expected calving date (prepartum), and 25 and 50 d postpartum of the studied cows in the spring season (March–May). Within 15–20 min. after collection, serum was separated by centrifugation (1600 × g/13 min) and stored at -20 °C until further analysis. *IGF-I* in the serum was measured using the *IGF-I*-ELISA Assay kit (Eagle Biosciences, Boston-Massachusetts, USA). The inter and intra-assay coefficients of variation were 6.8% and 7.4%, respectively, and the sensitivity was 3.3 ng/mL.

### DNA isolation, amplification, manipulation and sequencing

#### DNA isolation

Total genomic DNA was isolated from the blood samples (n = 1000) of the selected experimental cows using a DNA isolation kit (Tiangen Biotech, Beijing, China). The DNA samples were separated by electrophoresis on 1.0–1.2% agarose in 0.5 × TBE buffer according to Sambrook and Fritsch [73] after adding 0.5 μg/ml ethidium-bromide for quality assessment purpose. The electrophoresis run was performed using apparatus with a power supply and visualized by an ultraviolet transilluminator and Gel-documentation system (Chemi.Doc™ XRS + with Image Lab™ Software, BIO-RAD, USA). The purity and integrity of DNA were appropriate, and the OD260/280 was 1.82.

#### Amplification and manipulation

The specificity of the PCR primers targeting the *IGF-I* gene (249 bp fragment, 5’-noncoding region of the bovine *IGF-1* gene) and *GH* gene (432 bp fragment, fragment from Intron 4, part of exon 4 and part of exon 5) were previously tested by Ge et al. [43] and Balogh et al. [61]**,** respectively. The primer sequences, amplified region and product size of *IGF-I* and *GH* genes are shown in Table 6. The primers were synthesized by (Shanghai-Sangon Biolo. Engin. Tech. & Ser. Co., Ltd). The amplification was performed using (Green-Super.mix, TaKaRa, Japan). The PCR conditions are shown in Table 6. The amplification was carried out using a Thermo-cycler Gene Amp 6700 (Applied Bio-system, USA) and the products were separated by electrophoresis on 0.8% agarose and visualized by UV trans-illuminator and gel documentation system (Chemi.Doc™ XRS + with Image Lab™ Software, BIO-RAD, USA).

**Table 6:** The sequences, amplified region and product size of primers and PCR conditions for *IGF-I* and *GH* genes

Genes	Primer sequence						Amplified region		Product size
***IGF-I***	**F:5’**-ATTACAAAGCTGCCTGCCCC-**3’** **R:5’**-ACCTTACCCGTATGAAAGGAATATACGT-**3’**						5’-noncoding region of the bovine *IGF-I* gene		249
***GH***	**F:5’**-CGGACCGTGTCTATGAGAAGCTGAAG-**3’** **R:5’**-GTTCTTGAGCAGCGCGTCGTCA-**3’**						A 449 fragment from Intron 4, part of exon 4 and part of exon 5		432
**PCR conditions**									
**Genes**	*Denaturation*		*Annealing*		*extension*		*Final extension*		*Number of cycles*
***IGF-I***	Sec	°C	Sec	°C	Sec	°C	Sec	°C	N
	60	94	**60**	**65**	60	72	300	72	35
***GH***	Sec	°C	Sec	°C	Sec	°C	Sec	°C	N
	300	94	45	**63**	30	72	300	72	35

#### Nucleotide sequence analysis

Automated DNA sequence analysis was carried out on both strands by the DNA sequencing service lab of the Korean Research Institute of Bioscience and Biotechnology with an ABI Prism 3100 apparatus for both *IGF-I* and *GH* genes. Database similarity searches were performed with the FASTA network service at the National Centre for Biotechnology Information (NCBI) (http://www.ncbi.nlm.nih.gov). Also, the resulting sequences were analysed using MEGA 11, and Blast 2.0 software to detect SNPs between sequences. The sequences were deposited in GenBank. Moreover, the results of endonuclease restriction were carried out using ***FastPCR*** (http://primerdigital.com/fastpcr.html). Analysis of translated protein of *IGF-I* and *GH* sequences of the tested cows was generated and manged by BioEdit V.7.7. (https://bioedit.software.informer.com/7.2) and GeneScan (http://hollywood.mit.edu/cgi-bin/genscanw_py.cgi) with a minimum ORF size of 20 and the start codon AGT.

#### Restriction-fragment-length-polymorphism (RFLP) and electrophoresis

The RFLP was used to detect genotyping differences between and within tested cows using the PCR of target genes. The PCR amplicons of the *IGF-I* (249 bp) gene were digested with *SnaBI* (Jena Bioscience, Germany) and of *GH* (432 bp) with *AluI* (Bio-search Technologies, USA) separately. Defining restriction sites, before digestion with restriction enzymes was achieved by the NEB cutter program (http://www.labtools.us/nebcutter-v2-0). The RFLP-PCR reaction volume was 25 μl, consisting of 12 μl H_2_O, 2 μl 10X *HaeIII *buffer (Jena Bioscience, Germany), 1 μl (5 unit/ul) restriction enzyme in addition to 10 μl amplified DNA. All reactions were incubated at 37 °C for 16 h. Twenty μl of each reaction were separated by electrophoresis on 2.5% agarose gel and visualized by UV trans-illuminator and gel documentation system (Chemi.Doc™ XRS + with Image Lab™ Software, BIO-RAD, USA).

### Statistical analysis

#### Analysis of variance (ANOVA) and duncan test

The analysis primarily consisted of two steps: analysis of variance (ANOVA) and post-hoc tests. First, ANOVA was performed to evaluate whether significant differences existed in the serum concentration of *IGF-I* among the three genotypes across the periparturient periods (20 days prepartum, 25 days postpartum, and 50 days postpartum). Also, Significant differences among means were tested using the Duncan test.

#### Normality assessment and genotype effects analysis

All milk yield, milk composition, reproductive traits, and the serum concentration of *IGF-I* variables were tested for normality by Shapiro–Wilk test from the UNIVARIATE procedure of SAS (SAS, 2009), and the results indicated that all data were distributed normally (W ≥ 0.90). The genotype effects were analyzed using the GLM procedure of SAS by adapting the following model:

1 $$Y_{ijklmnop}=R_i+F_j+G_k+A_l+S_m+L_n+C_o+e_{ijkmnop}$$

where **Y**_**ijklmnop**_ is the mean value of the variable; **R**_**i**_ is the random effect of sire (i = 1,…..100 +), **F**_**j**_ is the fixed effect of the *IGF-I* genotype (j = 1,…., 3), **G**_**k**_ is the fixed effect of the *GH* genotype (k = 1 and 2), **A**_**l**_ is the fixed effect of the calving year (l = 2016, 2017 and 2018), **S**_**m**_ is the fixed effect of the calving season (m = 1,…. 4), **L**_**n**_ is the fixed effect of the parity (n = 1,2,3,4), **C**_**o**_ is the fixed effect of the milk production levels category (m = low, medium, high) and **e**_**ijklmnop**_ is the residual error. Significant differences among means were tested using the Duncan test.

#### Genetic indices and equilibrium analysis

The genetic indices of the studied animals: Heterozygosity (*H*_*o*_) and Heterozygosity expected (*H*_*E*_) were calculated according to Nei’s methods [74, 75]. Also, the Hardy–Weinberg equilibrium (HWE) was determined using Michael H. Court’s (2005–2008) calculator [76].

### Supplementary Information

Supplementary Material 1.

## Data Availability

The datasets generated and analyzed in this study are accessible in the Genbank repository with the accession numbers; *** NCBI Accession no.:***MH156810.1* (https://www.ncbi.nlm.nih.gov/nuccore/MH156810.1), *** NCBI Accession no.:***MH156811.1* (https://www.ncbi.nlm.nih.gov/nuccore/MH156811.1), *** NCBI Accession no.:***MH156812.1* (https://www.ncbi.nlm.nih.gov/nuccore/MH156812.1). Additional relevant information and results can be found in this manuscript and its supplementary files, including Figures, Tables, and Supplementary Figures.

## Abbreviations

*IGF-I*: Insulin-like Growth Factor-I gene
*GH*: Growth Hormone gene
SNPs: Single Nucleotide polymorphisms
HF: Holstein–Friesian
SCC: Somatic Cell Count
LP: Lactation length
DPRY: Dry period length
TMY: Total milk yield
305d-MY: Adjusted milk yield
% fat: Fat percentage
% protein: Protein percentage
EC: Electrical conductivity
pH: Acidity
DOPN: Days open
CI: Calving interval
GL: Gestation length
FPE: First postpartum estrus
NI: Number of inseminations
