# Supplementary material for: IGF-I and GH Genes polymorphism and their association with milk yields, composition and reproductive performance in Holstein–Friesian dairy cattle
Source: BMC Vet Res. 2024 Aug 2;20:341. doi: 10.1186/s12917-024-04188-4 (PMC11295711; doi:10.1186/s12917-024-04188-4)
Supplement: Supplementary file 1 — Supplementary Material 1. [file 12917_2024_4188_MOESM1_ESM.pdf]

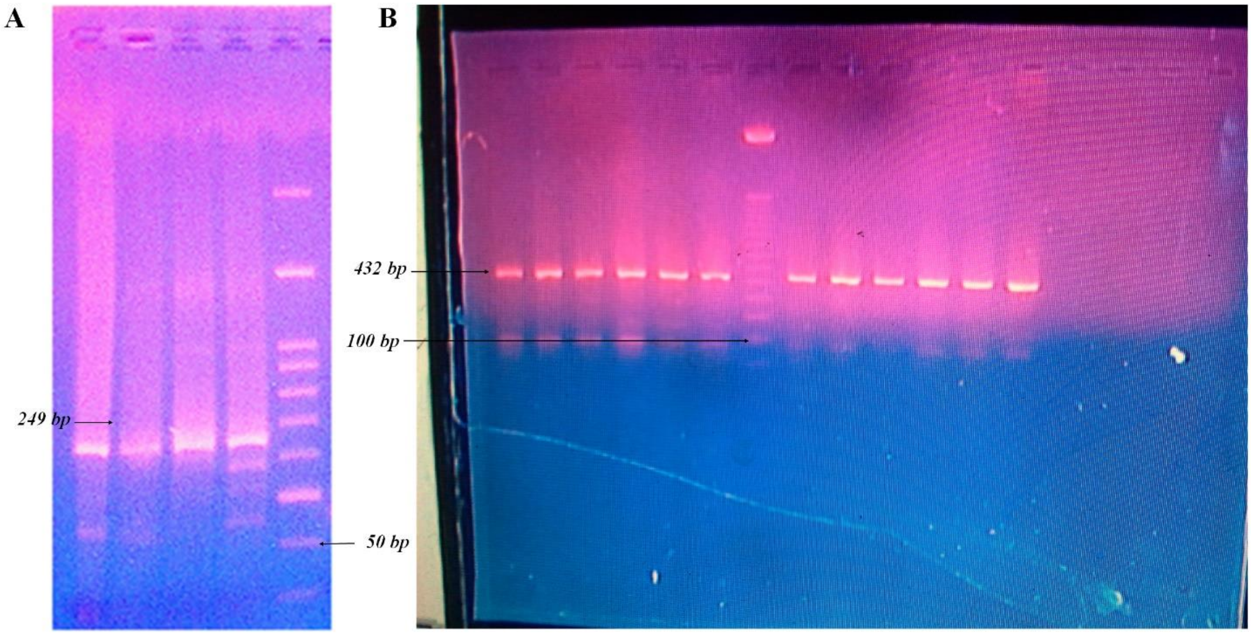

Fig. S1. **A)** PCR amplification of the 5’-noncoding region of the bovine *IGF-I* gene from Holstein Frisian cattle. M, 50 bp DNA ladder. **B)** PCR amplification of *GH* gene fragment from Holstein Frisian cattle. M, 100 bp DNA ladder.

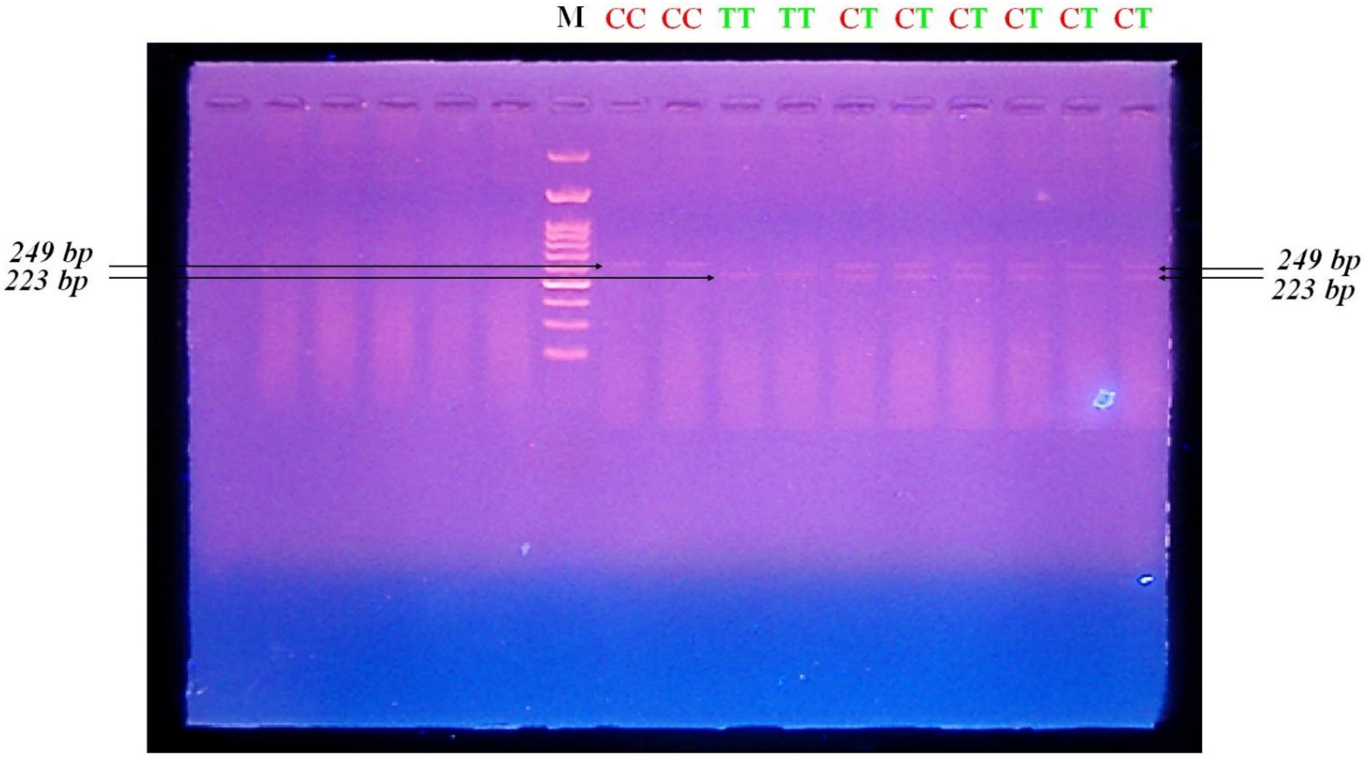

Fig. S2. Agarose gel electrophoresis showing RFLP-*SnaBI* restriction pattern of Holstein Frisian cattle in 5'-noncoding region of the bovine *IGF-I* gene Lane M: 50-bp ladder marker. Lanes 1 and 2: Homozygous CC; genotype non-digested PCR product (249 bp). Lanes 3 and 4: Homozygous TT genotype with 2 restricted fragments at 223 and 26 bp. Lanes 5 and 6: heterozygote CT genotype with 3 restricted fragments at 249, 223 and 26 bp. The restriction fragment with size 26 bp has not been seen on the gel,

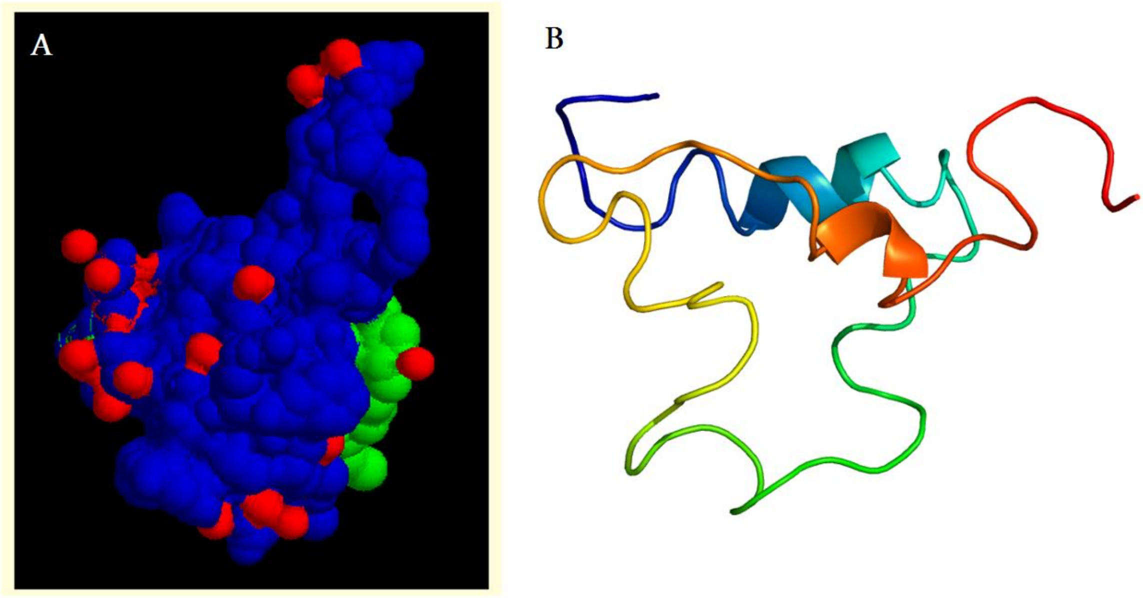

Fig. S3. 3D model of *IGF-I* (A). Structure of the *IGF-I* protein. Based on PyMOL rendering of PDB 1bqt (B).

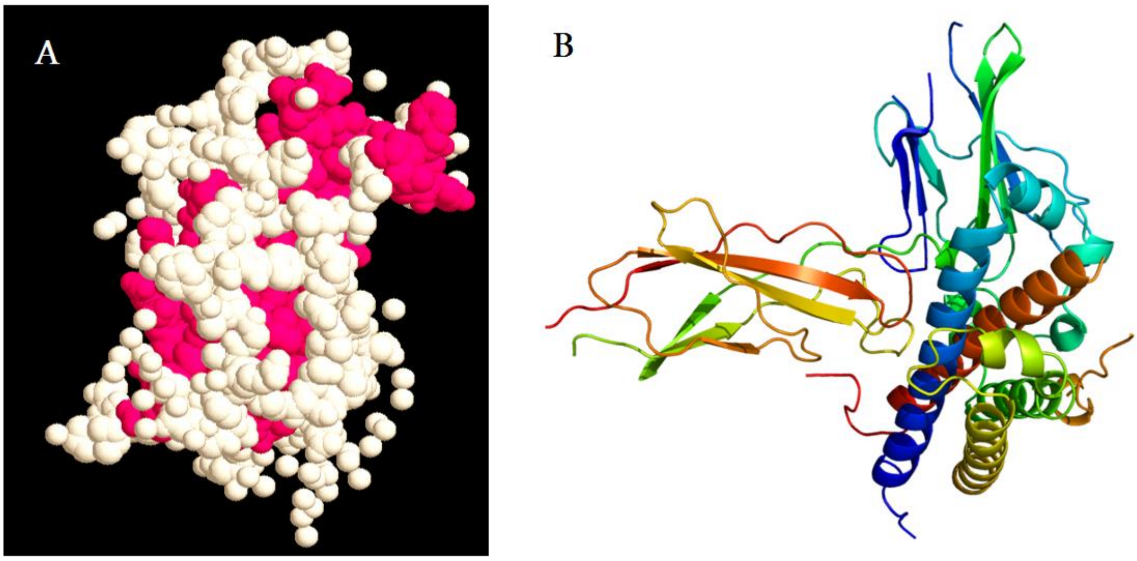

Fig. S4. 3D model of GH (A). Structure of the GH protein (B).

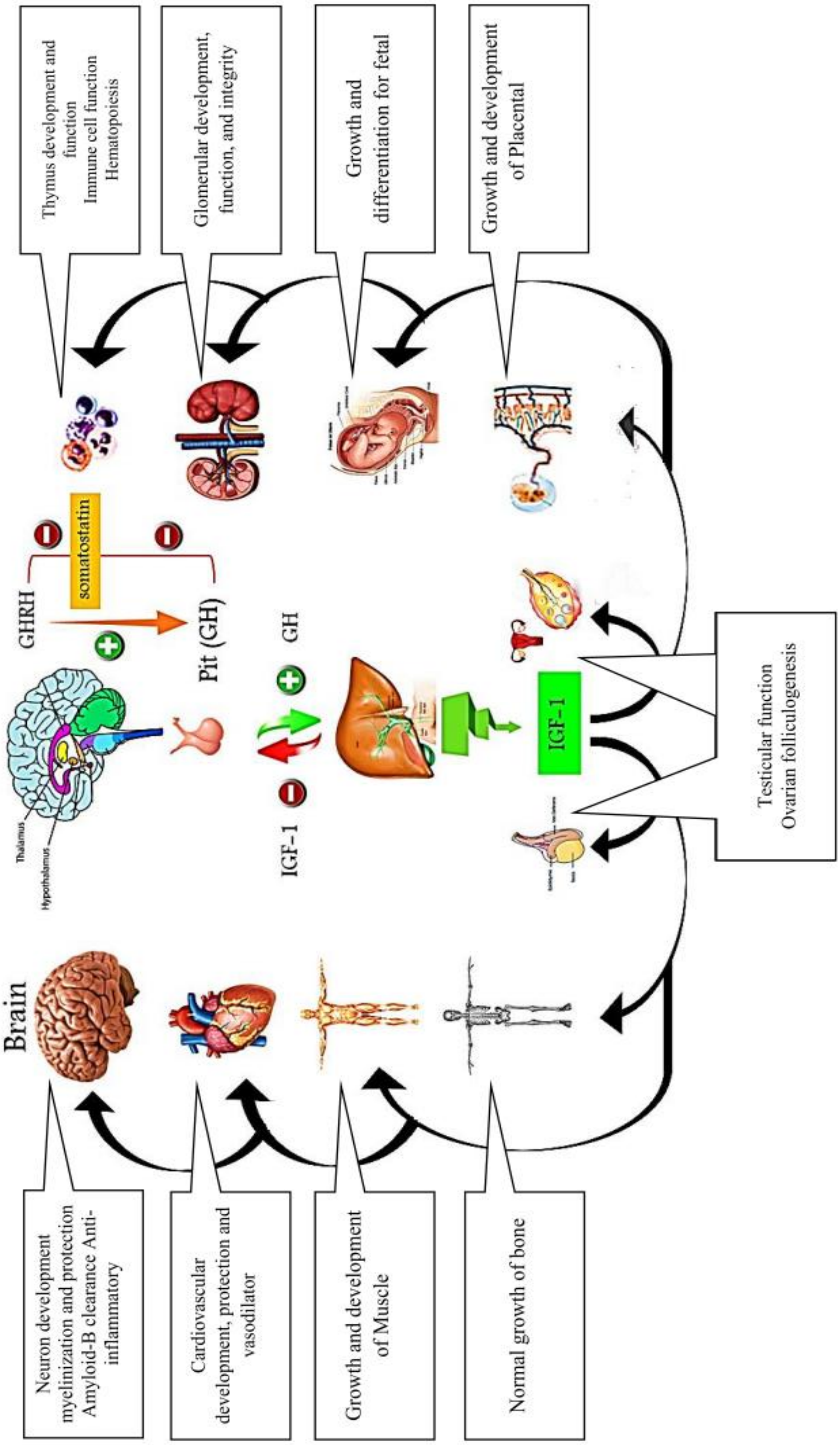

Fig. S5. Model of Growth hormone &Insulin Growth Factor-I (*GH/IGF-I*) axis and its target organs. Feedback mechanism induced by *IGF-I* regulates *GH/IGF-I* axis: *IGF-I* inhibits *GH* gene expression by stimulating somatostatin secretion. Growth hormone-releasing hormone (*GHRH*) stimulates *GH* secretion, which stimulates *IGF-I* secretion.

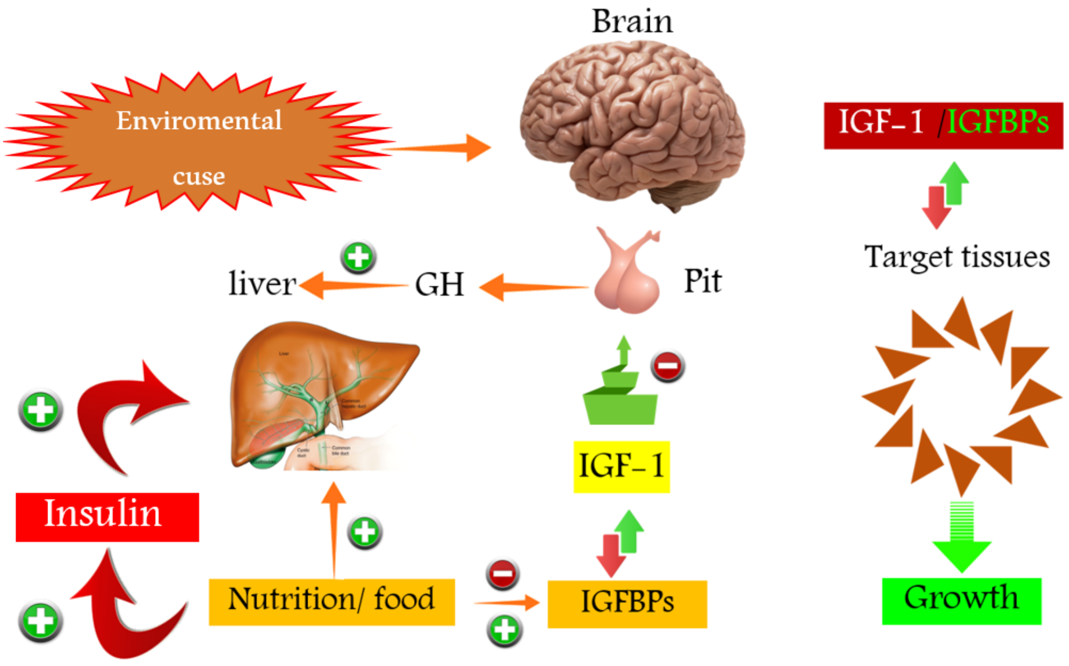

Fig. S6. Model of *GH-IGF-I* system controls. Growth hormone (*GH*) and insulin-like growth factor-I (*IGF-I*) are major regulators of postnatal metabolism and growth. Anything that increases growth hormone will increase *IGF-I*.

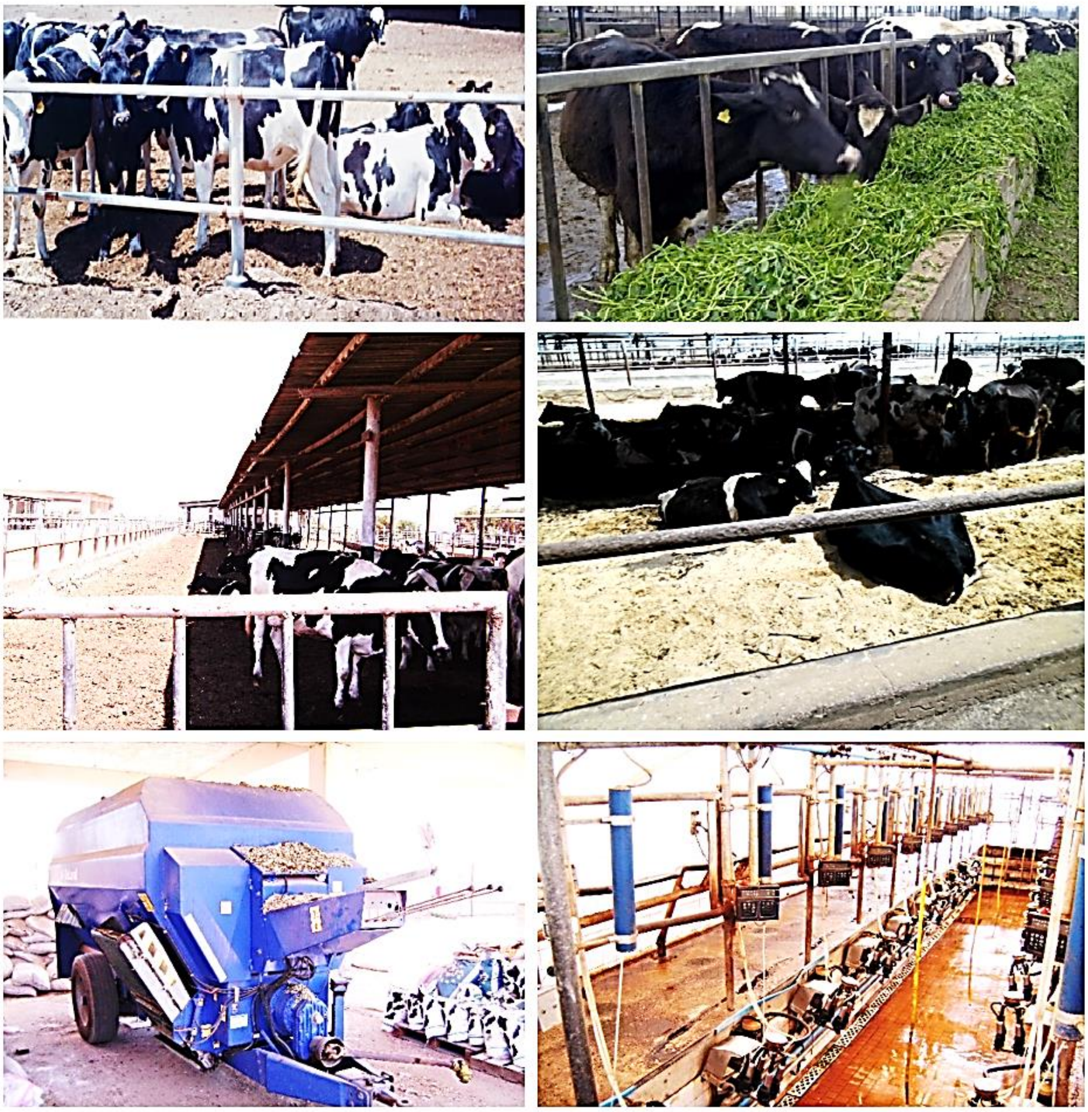

Fig. S7. Management practices in the dairy farm.
